# Evidence of microalgal isotopic fractionation through enrichment of depleted uranium

**DOI:** 10.1038/s41598-019-38740-2

**Published:** 2019-02-13

**Authors:** Beatriz Baselga-Cervera, Camino García-Balboa, Victoria López-Rodas, Marta Fernández Díaz, Eduardo Costas

**Affiliations:** 1 Department of Animal Science (Genetics), School of Veterinary Medicine, Complutense University, Madrid, 28040 Spain; 2 CIEMAT (Centro de Investigaciones Energéticas, Medioambientales y Tecnológicas), Madrid, 28040 Spain

**Keywords:** Environmental impact, Limnology, Nuclear fuel

## Abstract

Resulting from the nuclear fuel cycle, large amounts of depleted uranium (DU) tails are piling up, waiting for possible use or final disposal. To date, the recovery of the residual ^235^U isotope contained in DU has been conducted only marginally by physical processes. Relative isotope abundances are often mediated by biological processes, and the biologically driven U isotopic fractionation has been previously identified in reducing bacteria. Our results indicate that the cells of two microalgal strains (freshwater *Chlamydomonas* sp. (ChlGS) and marine *Tetraselmis mediterranea* (TmmRU)) took up DU from the exposure solutions, inducing U isotopic fractionation with a preference for the fissile ^235^U isotope over ^238^U. The n(^235^U)/n(^238^U) isotopic fractionation magnitudes (δ^235^) were 23.6 ± 12.5‰ and 370.4 ± 103.9‰, respectively. These results open up new perspectives on the re-enrichment of DU tailings, offering a potential biological alternative to obtain reprocessed natural-equivalent uranium. Additionally, the findings present implications for identifying biological signatures in the geologic records.

## Introduction

Motivated by the world’s continuously growing demand for energy and climate change becoming increasingly apparent, more efficient use of energy resources and diversification from fossil fuels (which are the principal cause of greenhouse gas emissions) has become a pressing need. Nuclear fission is an important alternative energy source to fossil fuels, as the energy conversion per gram of fuel is much higher and the carbon footprint is much lower. Moreover, compared to renewable alternative energies, nuclear power produces more energy, often at lower costs^1^. Nuclear electricity generation capacity in 2016 was 2.476 million GWh, approximately 11.5% of the total energy demand worldwide^2^. Therefore, nuclear power plays a key role in the gradual replacement of fossil fuels towards sustainable resources as part of the energy mix^3^: “*We need nuclear power as a bridge toward a post-fossil-fuel future*” (Professor Steven Chu, a Nobel Prize-winning physicist). Nuclear power generation is projected to increase and prevail to meet the future energy needs as a necessary stepping-stone towards a “clean” energy.

The basic nuclear fuel is initially uranium (U), whose consumption has been increasing rapidly, prompted by the nuclear energy demand. However, U resources on land are not unlimited^4^. The world´s present measured resources (5.7 Mt U) estimations are enough to last for the next 90 years^5^. Therefore, new viable sources of uranium are being sought. For example, extraction from seawater is gaining attention as an almost inexhaustible U fuel source^4,6^. Re-enrichment of depleted uranium (DU) is another secondary U fuel source.

Natural U consists of two main isotopes of interest: the fissile ^235^U (0.71 atomic %) and the fertile ^238^U (99.27 atomic %), along with the decay product ^234^U (0.0055 atomic %). In most cases, natural U needs different levels of enrichment depending on applications requirements (nuclear fuel range between a 3 and 5 atomic % of ^235^U^7^). The foundations of the nuclear fuel cycle are to obtain critical mass ‒ that leads to a sustained nuclear chain reaction ‒ of a fissionable nuclide in the nuclear fuel, such as the fissile ^235^U. One of the main hot spots in the nuclear fuel cycle is the tens of thousands of tons of DU tails (U resulting from the enrichment process, typically with ~0.0025-0.003 of ^235^U) sitting around with medium and low radioactive activity that must be confined with security in an already battered planet. For every ton of natural U enriched, approximately 130 kg of enriched fuel (~3.5 atomic % of ^235^U) is produced, and the balance is DU tails. The estimated world U requirements for 2017 are 65,014 Ut (76,671 t U_3_O_8_)^8^, with their subsequent major proportion resulting in DU. Annually, more than 50,000 tons of this byproduct swell the already substantial stockpiles only in U.S., Europe and Russia^9^. Even though DU has several uses, both civilian (i.e., radiation shielding of medical equipment or ballast in aircrafts) and military (particularly in ammunition), the estimated world’s stock is approximately 1.6 million tons of DU.

After processing, the by-product DU tails present the unique feature that they may be reprocessed and recycled to provide fresh nuclear fuel and reduce the volume of low-level wastes^10^. Several incentives of re-enrichment ‒ mainly in Russia^11^ and more recently by the U.S. Department of Energy ‒ have been put forward to recover the residual ^235^U contained in the DU and produce uranium with ^235^U natural contents (0.71 atomic %). However, current re-enrichment technology is only economically viable in centrifuge enrichment plants with spare capacity and low operation costs, and it involves high energy consumption and the associated CO_2_ emissions^12^. New technological developments pursuing a significant reduction of the environmental impact and greater U recycling-reprocessing would be desirable objectives considering the continuous increase of energy demand and the pressures upon energy sustainability^13^.

U is a ubiquitous element, present at significant amounts in the Earth’s crust^14^. U is not biologically linked with any type of life, yet various mechanisms through which U is biotically processed are common in the environment, for instance: biosorption, bioaccumulation, biomineralization, and biotransformation^15–19^. Consequently, microbial communities can also have dramatic effects on U mobilization/immobilization^20,21^. However, the study of *in vivo* and biologically mediated U isotope fractionation constitute research areas still to be explored. Traditionally, natural ^235^U and ^238^U variability, i.e., differential isotopic behaviours, has gone unaddressed and is assumed invariant owed to the small relative differences in mass of the isotopes^22,23^. Driven by the advent of technological advances in analytical measurements, the growing field of isotopic fractionation revealed considerable variations of U isotope ratios in natural settings (e.g., ores, granites, corals, seawater^22–24^). Therefore, significant U isotopic fractionation might take place at the Earth´s surface^22^ and represent a powerful tool in environmental, geological, marine, life and energy sciences. Biological U isotopic fractionation in nature has been linked to bacteria adept at inducing U(VI) biotic reduction^7,25–27^, as it was recently found that the redox reaction is responsible for the isotopic fractionation and is not related to the U uptake inside the cells^26^. Biologically U(VI) reduction studies resulted in the accumulation of ^238^U in the reduced product, except Rademancher *et al*.^27^ found ^235^U enrichment in the resultant U(IV). In another framework, human neuron-like cells *in vitro* achieved an isotopic fractionation of natural U with preferential intracellular uptake of ^235^U isotope^28^.

Hence, natural physicochemical processes leading to isotopic fractionation, both mass dependent and independent^29,30^, might take place during U uptake by the cells. This raises the question of whether different microalgal species give rise to U isotopic fractionation. Recently, evidence of U fractionation has been obtained during *Chlamydomonas* cells’ U uptake in a U acid mine drainage medium, suggesting a ^235^U enrichment^31,32^. Here, we have studied the U isotopic fractionation during DU uptake in two marine and freshwater Chlorophyta strains. As enrichment of the fissile ^235^U is expected in the cellular pellet, DU was used to address the potential in U reprocessing. Changes in the ^235^U/^238^U ratios of the extracellular and cellular U were investigated for 24 days in an extremophile *Chlamydomonas* (ChlGS strain) isolated from a U mining pond and a marine *Tetraselmis* (TmmRU strain). These results represent a potential tool for U recycling and reprocessing and may entail implications in the study of U isotopes in natural samples.

## Results

We performed two independent experiments, each with one of the strains of interest, the *Chlamydomonas* (ChlGS) and *Tetraselmis* (TmmRU) Chlorophyta strains, in different media supplemented with DU. ChlGS is an extremophile isolated from an acid U mine tailings pond, tolerant up to 25 mg U L^−1^ and other metals, and artificially selected for U uptake^33^. The isotopic ratio n(^235^U)/n(^238^U) with a value of 0.007375 ± 0.000013 found in the mine water (see Supplementary Fig. S1) was far above the consensus natural abundance ^235^U/^238^U [0.007198-0.007202]^34^, suggesting a possible enrichment process in the U mine pond. Conversely, TmmRU is a seawater strain, only previously exposed to naturally occurring trace U^35^ and subsequently selected for U tolerance. ChlGP cell replicates were exposed to 4 mg L^−1^ DU ~ 0.0050 atomic ^235^U freshwater stock solution and TmmRU to 2 mg L^−1^ DU ~ 0.0022 atomic ^235^U marine stock solution. The analytical procedure and sample resin purification validation were accomplished by the analyses of procedure control solutions (certified IRMM-053 material) between samples during the measure sessions to correct the bias induced during the inductively coupled plasma mass spectrometry (ICP-MS) measurements. The average n(^235^U)/n(^238^U) values found in the control solution was 0.007112 ± 0.000024 (σ, n = 34) (Fig. S2), these values were used to calculate the discriminatory factor of each sample (for definition, see SI Material and Methods).

**Figure 1:**
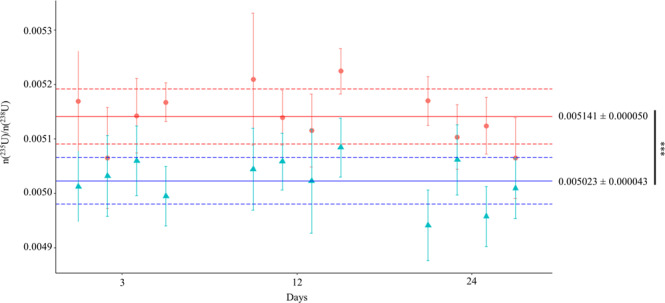
Freshwater ChlGS strain bioassay n(^235^U)/n(^238^U) (%) ratios and SD values over time. Cellular pellets (red circles) and supernatants (blue triangles) isotopic ratios were analysed by independent assays in groups of four at 3, 12, and 24 days. Solid lines correspond to the mean, and dashed lines correspond to the reproducibility (σ, n = 12), of the n(^235^U)/n(^238^U) values in the independent experiments for the cellular pellet (red lines) and supernatant (blue lines). Significant differences in the n(^235^U)/n(^238^U) values of supernatants and pellets appeared in the data according to a *t-*test (t = 6.17, df = 21.49, ^***^P < 0.001) (software package R version)^69^.

**Figure 2:**
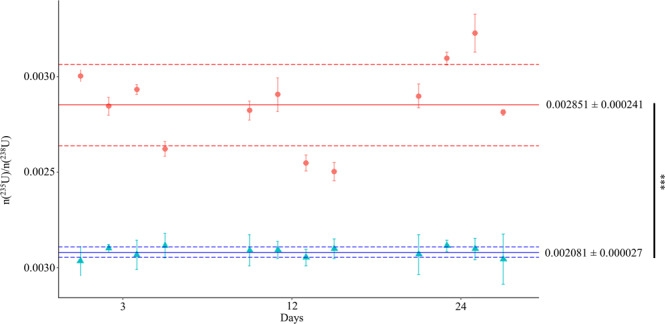
Marine TmmRU strain bioassay n(^235^U)/n(^238^U) ratios and SD values over time. Red circles and blue triangles represent the n(^235^U)/n(^238^U) ratios for the cellular pellets and supernatants, respectively, of independent assays analysed at 3, 12 and 24 days. Solid red and blue lines depict the mean n(^235^U)/n(^238^U) values obtained for the cellular pellet and supernatant, respectively, in 12 independent experiments. Dashed lines depict the reproducibility (σ, n = 12). Cellular pellets and supernatant values were significantly different according to the Wilcoxon test (W = 0.82, ^***^P < 0.001) (software package R version)^69^.

Within each bioassay, twelve independent trials were conducted, and the cellular pellet incorporated from 6.1 to 78.5 µg U for ChlGS and from 0.57 to 3.22 µg U for TmmRU (Table 1). ChlGS cells exhibited an increased U uptake performance until day 24, virtually the entire U amount in dissolution. Conversely, TmmRU cells incorporated 1.5–5% of the U present in the exposure solution. DU stock solutions were isotopically characterized before the exposition, and the average n(^235^U)/n(^238^U) ratio values found for the stock marine and freshwater solutions were 0.005058 ± 0.000083 (σ, n = 3) and of 0.002172 ± 0.000023 (σ, n = 3), respectively. The n(235U)/n(238U) ratios were determined in the cellular pellets and the extracellular solutions for each independent trial at different times: 3, 12 and 24 days (Figs 1, 2 and Table 1). The IRMM-053 material bracketed the samples for mass bias correction. No significant difference in the n(^235^U)/n(^238^U) ratio between days was found in either bioassay (*P* > 0.5).

**Table 1:** Results from the U isotopic fractionation bioassays in *Chlamydomonas* strain (ChlGS) and Tetraselmis strain (TmmRU). ¥δ235(‰) values were calculated of the respecting sample with the obtained n(235U)/n(238U) ratio of the cellular pellet relative to the supernatant.

Time (days)	Replicates	N° cells ± SD per mL (*10^6^)	U mass in cells pellet (µg)	^235^U/^238^U ratio ± SD (x10^−2^)		δ^235^(‰)^¥^
				Pellet	Supernatant	
***Chlamydomonas*** **(ChlGS)**						
3	ChlGS1	2.030 ± 0.255	13.4	0.5196 ± 0.0092	0.5012 ± 0.0065	31.2604
	ChlGS2	2.027 ± 0.228	8.6	0.5056 ± 0.0092	0.5032 ± 0.0074	6.5540
	ChlGS3	2.143 ± 0.201	8.1	0.5142 ± 0.0047	0.5059 ± 0.0064	16.3619
	ChlGS4	2.200 ± 0.330	6.1	0.5167 ± 0.0035	0.4994 ± 0.0054	34.6081
Mean ± SD	n = 4	2.100 ± 0.857	9.1 ± 3.1	0.5136 ± 0.0049	0.5025 ± 0.0028	22.196 ± 13.1012
12	ChlGS5	9.730 ± 0.193	31.0	0.5210 ± 0.0122	0.5044 ± 0.0076	32.8088
	ChlGS6	6.350 ± 0.880	17.6	0.5139 ± 0.00503	0.5058 ± 0.0052	16.0852
	ChlGS7	7.756 ± 0.754	15.1	0.5116 ± 0.0067	0.5022 ± 0.0095	18.6623
	ChlGS8	8.216 ± 0.556	21.1	0.5224 ± 0.0041	0.5084 ± 0.0054	27.6579
Mean ± SD	n = 4	8.013 ± 1.392	21.2 ± 7	0.5173 ± 0.0053	0.5023 ± 0.0025	25.5410 ± 9.51
24	ChlGS9	11.576 ± 0.636	78.5	0.5170 ± 0.0044	0.4941 ± 0.0065	46.3137
	ChlGS10	11.840 ± 0.866	45.1	0.5103 ± 0.0059	0.5061 ± 0.0064	8.2685
	ChlGS11	10.153 ± 0.105	63.3	0.5124 ± 0.00502	0.4957 ± 0.0056	33.7231
	ChlGS12	12.540 ± 0.749	72.9	0.5065 ± 0.0074	0.5009 ± 0.0055	11.1615
Mean ± SD	n = 4	11.527 ± 1.002	64.9 ± 14.6	0.5116 ± 0.0044	0.492 ± 0.0055	24.8667 ± 18.2733
***Tetraselmis*** **(TmmRU)**						
3	TmmRU1	0.494 ± 0.106	1.325	0.3002 ± 0.0033	0.2033 ± 0.0078	476.6755
	TmmRU2	0.467 ± 0.849	2.800	0.2844 ± 0.0047	0.2100 ± 0.0021	354.0278
	TmmRU3	0.610 ± 0.152	1.500	0.2932 ± 0.0025	0.2067 ± 0.0077	418.7661
	TmmRU4	0.352 ± 0.317	3.225	0.2621 ± 0.0037	0.2114 ± 0.0066	239.5483
Mean ± SD	n = 4	0.481 ± 0.105	2.212 ± 0.942	0.2850 ± 0.0166	0.2079 ± 0.0036	372.2544 ± 101.66
12	TmmRU5	1.280 ± 0.248	0.725	0.2824 ± 0.0082	0.2089 ± 0.0082	350.5646
	TmmRU6	1.389 ± 0.215	1.500	0.2906 ± 0.0088	0.2092 ± 0.0044	388.9685
	TmmRU7	1.198 ± 0.537	2.000	0.2547 ± 0.0041	0.2051 ± 0.0044	241.6516
	TmmRU8	1.206 ± 0.150	2.550	0.2502 ± 0.0048	0.2098 ± 0.0052	192.3654
Mean ± SD	n = 4	1.268 ± 0.088	1.693 ± 0.775	0.2694 ± 0.020	0.2082 ± 0.0021	305.1832 ± 76.209
24	TmmRU9	1.186 ± 0.449	1.025	0.2898 ± 0.0062	0.2068 ± 0.0103	401.1168
	TmmRU10	1.203 ± 0.571	0.575	0.3094 ± 0.0033	0.2113 ± 0.0032	464.6819
	TmmRU11	1.180 ± 0.112	1.125	0.3228 ± 0.0099	0.2096 ± 0.0057	540.0176
	TmmRU12	1.346 ± 0.977	0.775	0.2812 ± 0.0013	0.2043 ± 0.0131	376.3938
Mean ± SD	n = 4	1.228 ± 0.078	0.875 ± 0.248	0.3008 ± 0.0188	0.2080 ± 0.0031	445.5525 ± 73.1369

The δ^235^ (U) values, equation (S3), of each independent experiment were calculated from the n(^235^U)/n(^238^U) ratio between the cellular pellet and the supernatant (Table 1). For both microalgal species tested during this investigation, we observed a significant shift in U isotopic composition towards lighter values of δ^235^. Magnitudes of U removal from the dissolution by cells uptake were of δ^235^(U) _pellet-supernatant_ ≈ 22.2‒25.5‰ (ChlGS) and 305.2‒445.6‰ (TmmRU). Meanwhile, both studies found microalgal pellets to be considerably enriched in the isotopically light ^235^U isotope, whilst the supernatants showed a depletion in ^235^U. This implies an isotopic fractionation towards a lighter composition in the cell pellet in comparison to the supernatant. Additionally, these differences represent a down-blending of the isotope ^235^U in the cellular adjoin media of the isotope ^235^U, which necessarily was consumed in the process (Table 1).

## Discussion

Our results demonstrate that the two Chlorophyta microalgal strains studied fractionate DU. Each strain displayed a different enrichment factor but both reflect a strong fractionation of the light ^235^U isotope in the cellular pellet. The marine strain TmmRU, despite presenting higher cell volume and organic weight^36,37^, presented a lower U uptake rate. However, the enrichment factor was significantly higher than in the freshwater ChlGS, despite being exposed to a lower ^235^U content DU (~0.0022 ^235^U). The similar U isotopic composition found in the cellular pellets of the different replicates of each strain, irrespective of the culture time and the total U incorporated by the cells, raises the issues of preferential U uptake paths. The pathways leading to U uptake by the cells are not well documented^18^, and the joint complexity derivate of the simultaneously of several processes^28,38^ ‒ redox reactions, ligand exchange, diffusion, adsorption ‒ make interpretations of U fractionation origin extremely complex. Furthermore, U speciation and redox state may influence the fractionation process. Whatever the route, as previously described by Baselga-Cervera *et al*.^39^, U is bound to the outer wall and transported across the cell wall and membrane, becoming distributed inside the cell. These data suggest that U carried by the cellular pellet accomplishes isotopic partitioning, resulting in ^235^U being concentrated by the cells and the surrounding environment being enriched in ^238^U.

Consistent with our results, the isotopic ratio n(^235^U)/n(^238^U) value found in the U mine water suggest a possible enrichment process. Only microbial life has been detected in this pond^39,40^, and therefore biomass does not pass to higher trophic levels. Thus, ^235^U, as one of the lighter isotopes of U, could preferentially enter cells. When cells break, U enriched in ^235^U might be liberated to the water. The remaining U enriched in ^238^U bound to the cell wall might sediment in the bed of the pond. Additionally, bacteria that might be present in this pond and can contribute to this result. Reductive bacteria can induce U fractionation; the reaction products (U(IV)) are enriched in ^238^U, rendering the residual dissolved U enriched in ^235^U^25,41^. Combined effects of different microorganisms may have led to this result.

Microbial isotopic behaviour in elements with higher mass numbers, such as U, is typically poorly studied compared to light elements because of the need for more sophisticated and precise analyses. Recent evidence in the field has demonstrated U isotopic fractionation mediated by bacteria and neuron-like human cells. Biotic reduction studies with metal-reducing bacterial isolates show an enrichment in the heavier ^238^U isotope into the solid U(IV) byproduct^26,42,43^ that is not dependent on microbial sorption. Conversely, the isotopic behaviour displayed by neuron-like human cells showed a preferential ^235^U incorporation^28^. Paredes *et al*.^28^ suggested two possible isotopic fractionation processes based on the enrichment direction and U bioaccumulation: a mass-dependent “zero-point energy” mechanism^29^ and the mass-independent “nuclear field shift”. In the case of our study, as the two processes are not mutually exclusive and operate in the same fractionation direction, we cannot determine the precise contribution of each mechanism proposed. High-precision determination of other U isotopes, such as ^234^U ‒ commonly concentrated during ^235^U enrichment ‒ could provide insights into the fractionation mechanism at the cellular level and the proposed biological preference for lighter isotopes, but it is complex due to the abundances limitation [natural ^234^U abundance is comprehended between 0.000050–0.000059]. Reported isotope fractionation ratios for ^235^U/^238^U during U reduction by bacteria have ranged from −0.31 to −0.99‰^26,42,43^, and 0.38 ± 0.13‰ in neuron-like line cells^29^. We found that the cellular pellet was enriched in ^235^U relative to the supernatant with DU by 23.6 ± 12.5‰ and 370.4 ± 103.9‰ for the ChlGS and TmmRU strains, respectively. This fractionation behaviour is consistent in its direction with that observed in neuron-like cells, but the fractionation factors are significantly higher. One potential application of the observed microalgae-induced U isotopic variation is for DU waste re-enrichment along the U fuel cycle for nuclear fission energy. The current DU stockpiles worldwide would render one-third of natural-equivalent U after several cycles of re-enrichment, reducing DU tail and U-mine production. Currently, only centrifuge separation and gaseous diffusion have operated at commercial scale, even though several enrichment processes have been demonstrated historically and in the laboratory^44–47^. Both enrichment processes present important drawbacks: water reactivity by-products rendering hazardous compounds highly corrosive, as well as significant amounts of energy consumption, considerable costs and generate DU as a low-level waste product^48^. Reprocessing U tails with current techniques, despite its potential, is not economically and energetically feasible and does not ease the problem of final tail disposition^49^. Particularly interesting would be to develop a viable biological process to enrich DU, recovering natural-equivalent U from tailings waiting for final disposal without high energy demand and the associated carbon footprint. Hypothetically, multiple stages of microalgal bioaccumulation of DU, cell harvesting and U resuspension into the next higher step would enrich the DU to the desired amount, up to the ^235^U natural content or even above. The predicted advantages of this biological process are the reduced cost and low energy requirements, turning DU in a resource that even the U-tail problem decreases only marginally. Further experimentation may resolve the expected value range for U fractionation mediated by microalgal bioaccumulation and the efficiency of the enrichment process. In addition, other microalgal species might display a different isotopic behaviour, considering the significant differences exhibited by the two Chlorophyta species.

Our findings might also have implications for the identification of biotic signatures using isotope tools to study ancient microbial life and understand global uranium flux. Significant uncertainties remain regarding isotopic signatures owing to the ambiguity in the interpretation of the signs. Development of analytical techniques has opened the possibility of studying small U isotope natural variation. Uranium is the heaviest element for which natural variations have been reported^50^. U isotopic fractionation in nature occurs, with opposite directions, in both anoxic/euxinic and oxic environments and can be associated with chemical transformations such as adsorption, speciation or redox chemistry^22^. Microorganisms probably have a part in the U cycling and deposit formation in nature^51–53^. The large isotope fractionation that occurs during microalgal U uptake suggests the role of microalgae in the conservative behaviour of U and a tight range of isotope composition in modern oceans. For geological implications, recent studies of biotic redox transformation of U with metal-reducing bacteria induced U isotopic fractionation enriching for ^238^U in the reaction products^25,26^, contradicting previous studies^27^ and consistent with environmental studies and U-reduced depositional samples^22,41,43,53,54^. U isotopic fractionation mediated by bacterial enzymatic reduction raises as a tangible biosignature in the rock record for specific metabolic groups and onto the timing emergence of specific metabolisms. In oxic sedimentary environments, sample fractionation occurs towards lighter isotope composition, and intriguingly, banded-iron formation samples present the lightest U composition studied^22^. The banded-iron formations’ lighter values could indicate microbial phototroph co-precipitation by adsorption of iron and U, supporting the previously suggested microbial implication^55^. Thus, the preferential accumulation of the fissile ^235^U isotope in sediments might be a proxy for the activity and presence of microalgae. U fractionation biological fingerprints in ancient sedimentary rocks would provide insights into ancient microbial activity and establish temporal constraints. Additionally, our findings might provide insights into the Oklo phenomenon on the assumption of a microbial contribution in the initiation of the natural nuclear reactors^40,56,57^. Several factors probably contribute to these unique chain reactions. Two thousand million years ago – the same time proposed for Oklo’s event^58–61^ – ^235^U made up approximately 3% of the natural U, a condition that makes possible the starting of a fission reaction. However, a U-rich mineral deposit needs to be formed to obtain a critical mass. Most likely, the presence of increasing oxygen in the Earth’s atmosphere enabled the U flux and subsequent concentration in U ore bodies^62^. The direction and magnitude of the observed microalgal U isotopic fractionation during bioaccumulation could support the biological hypothesis of the origin of the natural reactors.

## Methods

### Microalgal species and culture conditions

We used two Chlorophyta, a freshwater environmental extremophile, *Chlamydomonas saelices nov*. sp. (strain ChlSG) isolated from a uranium mining pond in Saelices el Chico, Salamanca, Spain (previously described in depth by García-Balboa *et al*.^39^ and Baselga-Cervera^31^), and the marine *Tetraselmis mediterranea* (Lucksch) R.E. Norris, Hori & Chihara (strain TmmRU) isolated in the east-coastal waters of Sardinia (Italy). Both strains were selected for U tolerance and placed in the Spanish Algae Bank (Spanish acronym BEA) with the access numbers BEA/D04-12 and BEA/IDA/0062 for ChlSG and TmmRU, respectively^63,64^. Strains were propagated asexually in cell culture flasks (Greiner; Bio-One Inc., Longwood, NJ, USA) with BG-11 medium (Sigma-Aldrich Chemie, Taufkirchen, Germany)) for ChlGS and Guillard’s F/2 medium (Sigma-Aldrich Chemie, Taufkirchen, Germany) for TmmRU (media were prepared according to the manufacturers’ directions). Strains were sub-cultured by serial transfers to fresh medium once every 20 days to ensure mid-log-exponential growth, and the axenicity was regularly tested. Culture conditions were continuous illumination at 80 µmol m^−2^ s^−1^ over the wavelength range from 400 to 700 nm and a temperature of 22 °C.

Prior to experiments, both microalgal cultures of ChlGS and TmmRU were analysed to ensure the absence of U before exposure to DU. No U was detected in either of the cultures.

### Exposure experiments to DU

Here, we studied the U fractionation behaviour of two microalgal species artificially selected for U recovery^63,64^: the freshwater extremophile *Chlamydomonas* (strain ChlGS) isolated from a well-studied U acid mine pond^31,39^ and the marine *Tetraselmis* (strain TmmRU) isolated from the Mediterranean sea only sparsely exposed to naturally occurring trace ^235^U. Two different sets of experiments were performed with two different strains ChlGS and TmmRU. In both bioassays, twelve replicates were established in cell culture flasks with 20 mL of depleted uranium medium with approximately 2 × 10 ^6^ cells and 0.5 × 10 ^6^ cells per mL for ChlGS and TmmRU, respectively. In the case of ChlGS, the depleted uranium medium consisted in a preparation of 4 mg L^‒1^ of U with an isotopic relation n(^235^U)/n(^238^U) of 0.005058 ± 0.000083 in bi-distilled water sterilized and enriched with BG-11. In the TmmRU bioassay, the depleted uranium medium consisted of a preparation of 2 mg L^‒1^ of U with an isotopic ratio n(^235^U)/n(^238^U) of 0.002172 ± 0.000023 in bi-distilled water sterilized and enriched with F/2. Our experiments were performed in aqueous systems, oxidizing conditions and below Σ pH 7–8, conditions were the mobile U(VI) is predominantly found as uranyl ions (UO^2+^_2_) or hydroxyl complexes^18,65,66^. pH was selected at the onset of the microalgal fractionation experiments to have predominantly soluble U species, so ligands of the cell walls would attract U cations though chemical sorption^67^. The U concentration for TmmRU was obtained from the dose-response curve and represented the higher dose before the IC_50_ values for growth inhibition. In ChlGS strain, the U concentration used was selected based upon previous U uptake experiments^33^. At different times along the cells’ growth curve (3, 12 and 24 days), four independent cultures were centrifuged at 4000 rpm for 15 minutes, and the two phases obtained (supernatant and pellet) of each replicate were frozen. Throughout the experiments, due to the media alkalization induced by the cells’ photosynthetic activity, some U precipitation could take place.

The experimental media were supplied by the Energy, Environmental and Technological Research Center (Spanish acronym CIEMAT). All the samples were sent to CIEMAT for isotopic analysis and total U analyses. The isotopic relation results were obtained by averaging the data obtained in three analyses.

### Uranium fractionation characterization

Algal pellets (mg) and filtered supernatants (5 mL) were treated by microwave acid digestion with 5 mL of 65% HNO_3_ and 30% H_2_O_2_ (4:1 v/v). For pellet samples, before microwave treatment, the sample-reagent mixture was sonicated. After digestion, the sample solution was evaporated almost to dryness, and the residue was dissolved to 5 mL with 3 M HNO_3_. One millilitre of this solution was brought to a final volume of 10 mL with MilliQ water to quantify the U by quadrupole-based ICP-MS (Q-ICP-MS) using the external calibration and internal standard method. The remaining volume was used for isotopic analysis after purifying U with UTEVA resin according to revised protocols reported by Carter *et al*.^68^ and Weyer *et al*.^22^, summarized as follows. The sample solution was loaded onto a previously washed and pre-conditioned column. The column was rinsed with 2 × 5 mL of 3 M HNO_3_, 6 mL of 9 M HCl and 5 × 3 mL of 5 M HCl. Uranium was eluted in 5 × 3 mL of 0.02 M HCl. The collected uranium fraction was evaporated and the residue re-dissolved in 2 mL of concentrated HNO_3_, and the uranium was again evaporated. This last residue was dissolved in HNO_3_ 2%. Matrix separation and U concentration were checked in the fractions collected by Q-ICP-MS (iCAP Q, Thermo Scientific). Procedural blanks of the entire sample treatment procedure, including digestion and purification, were performed in every sample batch.

Due to the high uranium content, purified samples were diluted with HNO_3_ 2% before isotope ratio measurements to a concentration that matched that of the isotopic standard IRMM-053.

Isotope ratios were determined in a double-focusing sector field ICP-MS (Element 2, Thermo Scientific) equipped with a single collector and using a desolvating system (Cetac Aridus II) for sample introduction. This unit stabilized the ion beam and provided a 5-fold enhancement in sensitivity (10 × 10^6^ cps per ng.g^-1^). Measurements were carried out under optimized conditions of the overall system to obtain the maximum accuracy and precision in isotopic measurements (for detail information of the procedure validation, see SI Material and Methods). For data acquisition, standard bracketing was used placing every sample between two isotopic standards (IRMM-053) and washing with HNO_3_ 2% before every sample and standard (see experimental settings in Supplementary Table S1).

Potential isotope fractionation due to chromatographic extraction was evaluated by measuring the ^235^U/^238^U ratio in the isotopic CRM as well as in the DU before and after passing through the column using the same elution protocol as in the samples.

## Supplementary information

Supplementary Material

Raw data

## Data Availability

All data generated or analysed during this study are included in this published article (and its Supplementary Information Files).
