# Correction: Evidence of microalgal isotopic fractionation through enrichment of depleted uranium

**DOI:** 10.1038/s41598-026-47799-7

**Published:** 2026-04-16

**Authors:** Beatriz Baselga-Cervera, Camino García-Balboa, Victoria López-Rodas, Marta Fernández Díaz, Eduardo Costas

**Affiliations:** 1 Department of Animal Science (Genetics), School of Veterinary Medicine, Complutense University, 28040 Madrid, Spain; 2 CIEMAT (Centro de Investigaciones Energéticas, Medioambientales y Tecnológicas), 28040 Madrid, Spain

Correction to: *Scientific Reports* 10.1038/s41598-019-38740-2, published online 13 February 2019

This Article contains errors, where the Data Availability section is incomplete. The Data Availability section has been revised to clarify the conditions for access of the algae strains deposited at the Spanish Algae Bank. The Data Availability section should read:

“All data generated or analysed during this study are included in this published article (and its Supplementary Information Files). The strains that support the findings are deposited at the Spanish Algae Bank (Spanish acronym BEA) with access numbers BEA/D04-12 and BEA/IDA/0062 for ChlSG and TmmRU, respectively. Restrictions apply to the availability of these strains, which were used under license for the current study (Universidad Complutense de Madrid owned patents WO2014087030A1 and ES2633038B2). Strains are available upon request and with permission of the Oficina de Transferencia de Conocimiento (OTC). Propiedad industrial e intelectual. Universidad Complutense de Madrid. otripat@ucm.es”

