# Supplementary material for: Evidence of microalgal isotopic fractionation through enrichment of depleted uranium
Source: Sci Rep. 2019 Feb 13;9:1973. doi: 10.1038/s41598-019-38740-2 (PMC6374374; doi:10.1038/s41598-019-38740-2)
Supplement: Supplementary file 1 — Supplementary Material [file 41598_2019_38740_MOESM1_ESM.docx]

# *Supplementary Material*

**Evidence of microalgae isotopic fractionation through enrichment of depleted uranium**

**Beatriz Baselga-Cervera^1^*, Camino García-Balboa^1^, Victoria López-Rodas^1^ Marta Fernández Díaz^2^ & Eduardo Costas^1^***

**Correspondence:** Corresponding Authors: [ecostas@ucm.es](mailto:ecostas@ucm.es); [bbaselga@ucm.es](mailto:bbaselga@ucm.es)

**SI Material and Methods**

Reagents and calibration

All reagents used were of Suprapur quality and, in the case of the acids (HNO_3_, HCl), sub-boiled in addition. Working solutions were prepared using > 18,2 MΩ·cm H_2_O from Milli Q water system (Millipore Corporation).

The analysis conditions were established following the manufacturer’s recommendations. The standard isotopic materials ^235^U (IRMM-050) and ^238^U (IRMM-053) were supplied by the Institute for Reference Materials and Measurements (GEEL, Belgium). IRMM-053 solution (⁓ natural U) with a certified value of 0.0072623 for n(^235^U)/n(^238^U) was used to correct for mass discrimination effects in all the isotopic analyses performed. IRMM-050 was occasionally used to evaluate the correction with another standard of different composition. Finally, it was decided to use IRMM-053 because its isotopic composition was closer to that of the samples measured. The ICP-MS measurement process induces a slight variation in the real isotopic composition of the samples. This bias is known as instrumental isotopic fractioning and is mainly due to the different behaviour of ions in the system according to their mass. The absolute value of the isotopic ratio (R_real_) in the ^235^U/^238^U ratio can be calculated by:

$R_{real = R_{measured}\times F_{d}}$(Eq. S1)

where R_real_ is the real value of the isotopic ratio and R_measured_ the isotopic ratio obtained by the instrument and F_d_ discriminatory factor.

The value of F_d_ was estimated for each sample, before each measurement, from a certified isotopic solution as follows:

$F_{d}=\frac{\left( \frac{{}_{-}^{235}U}{{}_{-}^{238}U} \right)_{certified}}{\left( \frac{{}_{-}^{235}U}{{}_{-}^{238}U} \right)_{measured}}$(Eq. S2)

Therefore, the biological isotopic enrichment was calculated as:

$\delta^{235} (‰)=\left[ \left( \frac{U_{p}^{235/238}}{U_{s}^{235/238}} \right)-1 \right]\times1000$(Eq. S3)

where $U_{p}^{235/238}$is the mean value of the pellet sample isotopic ratio and $U_{s}^{235/238}$ is the mean value of the isotopic ratio found in the supernatants.

Analytical procedure validation

U content in both microalgal strains, *Chlamydomonas* (ChlGS) and *Tetraselmis* (TmmRU), was measured before the experiments to ensure the absence of U in the culture suspension (for ChlGS, it was isolated from a U mine pond). Analyses confirmed that the culture suspensions of both microalgae did not contain U.

In the same manner, to control the U contribution due to the treatment of the samples prior to the isotope ratio measurement, procedural blanks of the total process, including digestion and the chromatographic stage, were performed in each batch of samples. The measured U in these procedural blanks was at the pg level (50-100) depending on the batch processed, whereas the concentration of U in all samples was of the order of micrograms.

The effectiveness and yield (U recovery) of the purification process was tested by analysing the collected U fractions by Q-ICP-MS. All the elements detected (E) were at trace levels (U/E > 10^3^). In addition, the same U fractions were passed to HNO_3_ medium and heated to dryness with the purpose of eliminating those molecules that could co-elute with the U as well as rests of resin dragged in the process. According to the measured U, the average recovery of the purification step was 95-100%.

Instrumental mass discrimination effects (mass bias), as well as matrix-dependent non-spectral mass discrimination effects, induced by changes in sample composition were considered as follows. Isotope ratios were measured in a double-focusing magnetic sector field ICP-MS with SEM single collector (Element2, Thermo) equipped with an Aridus II Desolvating Nebulizer System (Cetac). Solutions of isotopic standard and samples were matched at a concentration allowing the measurement of both isotopes in the same detector mode, thus avoiding inaccuracies from cross-calibration between detector modes. Mass bias was corrected by means of the sample standard bracketing method using the certified isotopic standard IRMM-053. The use of external standardization allowed us to determine the mass bias in the same masses as the analyte ratio and with approximately the same abundances. Nonspectral matrix effects were evaluated by means of internal standards (In and Lu) incorporated continuously in all samples (problem samples, isotopic standard, and blanks) throughout the analysis. It was found that the variations of the internal standards had no relationship with the class of the sample (isotopic standard or problem sample).

To rule out the possibility of U fractionation during the sample preparation, a certified isotopic standard, as well as the DU standard, were subjected to the same treatment as samples (digestion and purification). At every stage, aliquots were taken to determine the ^235^U/^238^U ratio. The measured isotopic ratios in all fractions did not differ statistically from the isotopic ratios measured in these same standards directly diluted in HNO_3_.

Procedural blank subtraction was done in the raw data and in each isotope since procedural blanks and samples could have different ^235^U/^238^U ratios.

**Supplementary Figures**

**Supplemental Figure 1.** n(^235^U)/n(^238^U) ratios and S.D. analytical values of U radionuclides in the U mine waters. The n(^235^U)/n(^238^U) values shown correspond to the IRMM-053 isotopic standard certified amount ratio (filled circles) and U mine water samples spiked with the IRMM-053 material (filled triangles).
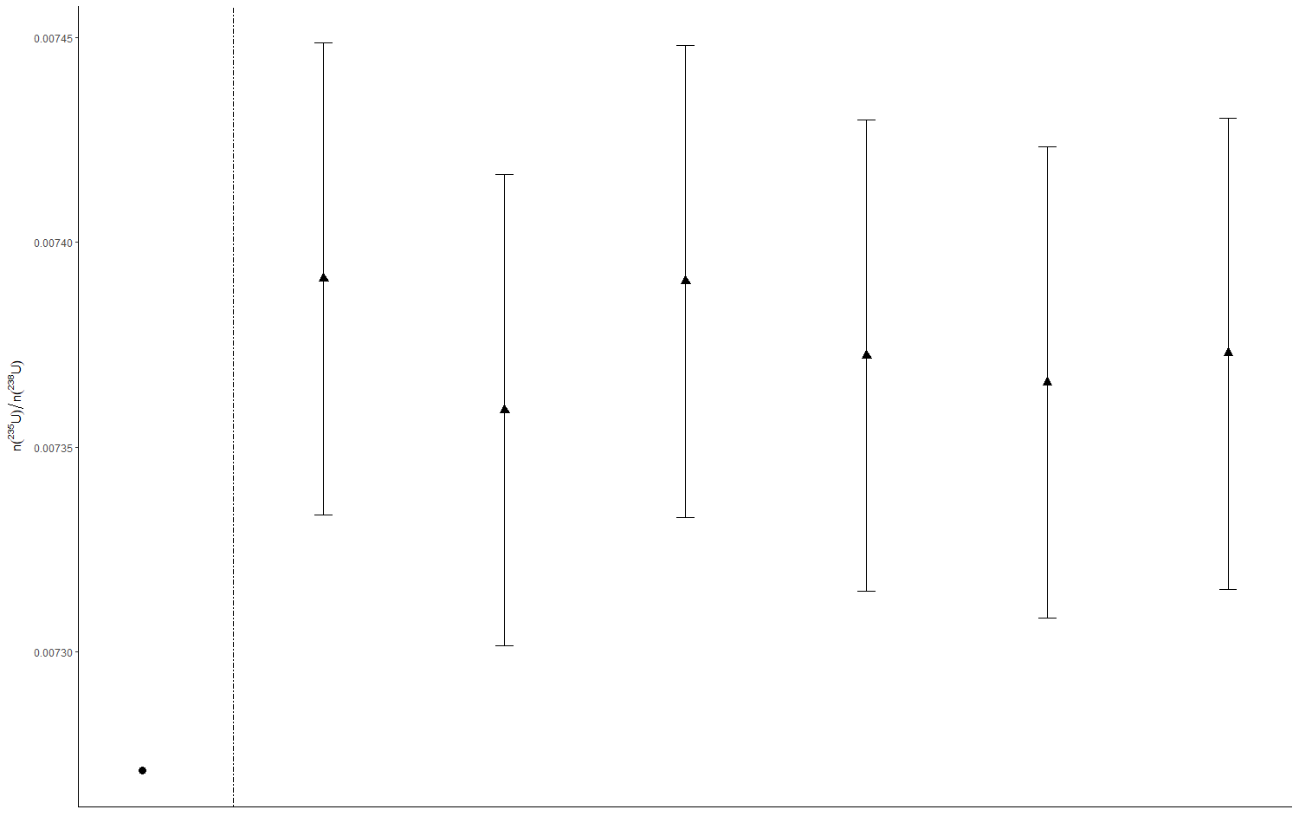


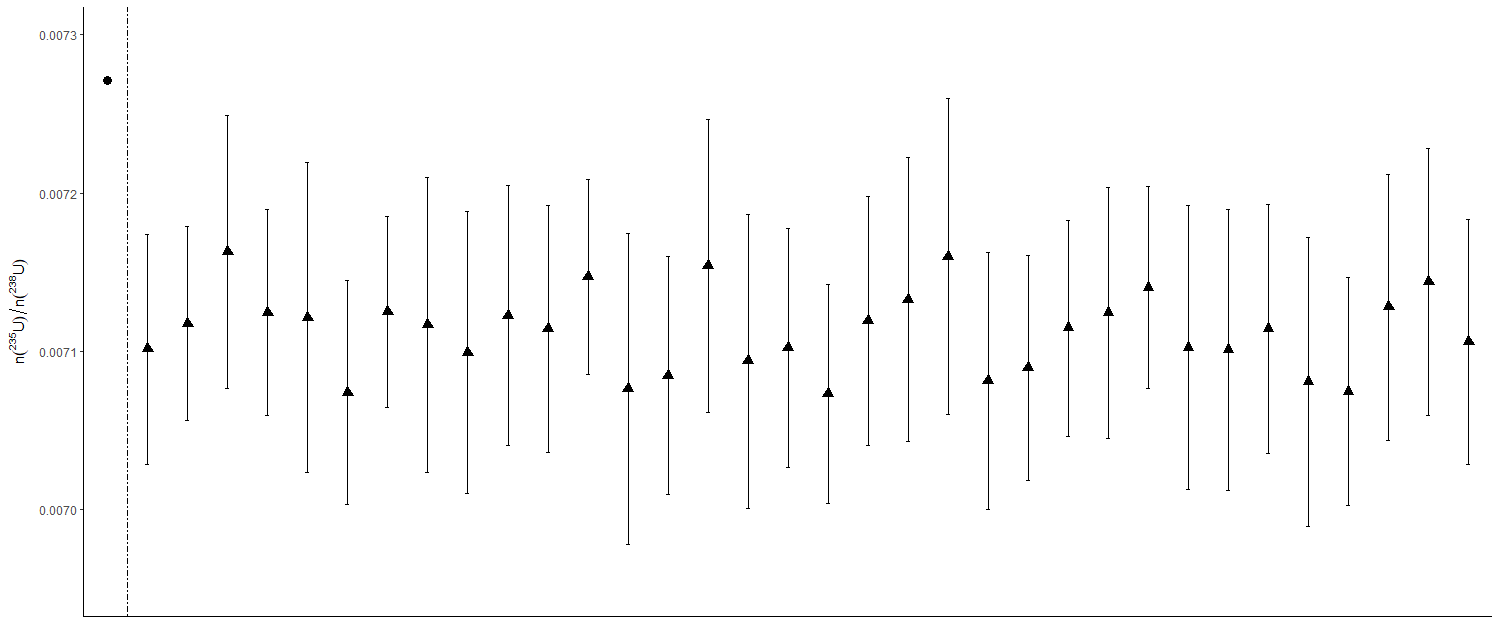
**Supplemental Figure 2.** n(235U)/n(238U) ratios ± 1SD measured for the IRMM-053 procedure control solutions. The n(235U)/n(238U) values shown correspond to the certified value (~0.007271, filled circles) and the analysed IRMM-053 control solutions interspersed with the experiment samples (filled triangles).

**Supplementary Tables**

**Supplemental Table 1.** Experimental Settings for the Element2 ICP-MS. Instrument configuration: Element 2 (Thermo) + Cetac Aridus II™ Desolvating System equipped with PFA nebulizer. The spectrometer was tuned (Ar gas flows, plasma RF power, lens voltages, mass calibration, etc.) before data acquisition. For measurement, every sample was bracketed by two isotopic standards, and a blank acting as a washing solution was measured before every sample and standard.

| **Operating parameters** | |
| --- | --- |
| ***Plasma*** |  |
| RF power | 1300-1330 W |
| Coolant Ar flow | 16 l min^-1^ |
| Auxiliary Ar flow | 1.1 l min^-1^ |
| ***Aridus II™ Desolvating System*** |  |
| Spray chamber temperature | 70 °C |
| Membrane Oven temperature | 160 °C |
| Ar Sweep Gas | 5.0 l min^-1^ |
| ***Data acquisition*** |  |
| Resolution m/Δm | 300 (low resolution) |
| Acquisition mode | Peak jumping* |
| Monitored isotopes | ^235^U – ^238^U |
| Acquisition mass window | 5% |
| Number of scans | 500 (5 runs, 100 passes) |
| Samples per peak | 300 |
| Detection mode | Pulse counting |
| Sensitivity for U | 10 x 10^6^ cps ppb^-1^ |

*Fixed magnetic field and variable electrostatic field (E-scan)
